# Validation of an original Behavioral Changes Scale on the Use of Digital Technologies During Social Distancing

**DOI:** 10.47626/2237-6089-2020-0173

**Published:** 2022-10-18

**Authors:** Mariana King Pádua, Anna Lucia Spear King, Lucio Lage Gonçalves, Hugo Kegler Santos, Douglas Rodrigues, Antônio Egidio Nardi

**Affiliations:** 1 Laboratório Delete – Detox Digital e Uso Consciente de Tecnologias Instituto de Psiquiatria Universidade Federal do Rio de Janeiro Rio de Janeiro RJ Brazil Laboratório Delete – Detox Digital e Uso Consciente de Tecnologias , Instituto de Psiquiatria , Universidade Federal do Rio de Janeiro (UFRJ), Rio de Janeiro , RJ , Brazil .; 2 Departamento de Estatística Instituto de Matemática e Estatística Universidade Federal Fluminense Niterói RJ Brazil Departamento de Estatística , Instituto de Matemática e Estatística , Universidade Federal Fluminense (UFF), Niterói , RJ , Brazil .

**Keywords:** Digital technologies, social distancing, human behavior, internet, COVID-19

## Abstract

**Introduction:**

The social distancing (SD) adopted during the coronavirus disease 2019 (COVID-19) pandemic has transformed the internet from a convenience into a necessity. The behavioral changes caused by isolation range from adaptation of consumption, work, and teaching routines to altered leisure options to occupy idle time at home. Such transformations can be positive, expanding use of digital technologies (DT), but they can also have serious future physical and emotional consequences if there conscious use of technological devices is lacking.

**Objectives:**

The study aimed to validate the Behavioral Changes Scale on the Use of Digital Technologies During Social Distancing (BCSDTSD), an instrument for assessing behavioral changes related to use of DT during SD.

**Method:**

Validation of the BCSDTSD in five phases: 1. construction of an initial scale with 10 questions; 2. evaluation of the questions by a panel of experts; 3. application to 1,012 volunteers via the internet; 4. statistical analysis of the results; and 5. preparation of the validated final version of the BCSDTSD. Data were analyzed using the *dplyr, psy* , and *paran* packages and the REdaS statistical program. Three statistical criteria were used in the factor analysis (FA).

**Results:**

FA confirmed that all 10 questions in the questionnaire should be maintained, confirming its robust construction, and Cronbach’s alpha demonstrated its internal consistency with a value of 0.725, which is satisfactory for first-application questionnaires.

**Conclusion:**

The BCSDTSD instrument was validated for assessment of behavioral changes related to the use of DT during SD.

## Introduction

Declaration of the new coronavirus disease 2019 (COVID-19) as a pandemic led several countries to adopt social distancing (SD) measures to slow the spread of the new disease. The objective of creating spatial distance ^1^ and maintaining safe distances between individuals prompted closure of schools, sports arenas, commercial establishments, and entertainment activities, among others. ^2^ Regulations and recommendations resulted in people’s isolation at home and limited their free movement, with freedom of work limited to frontline workers in the fight against the crisis. ^1^

Prior to the pandemic, daily life had already become more dependent on interaction with digital technologies (DT) (computers, smartphones, tablets, etc.), ^3^ but SD changed cyberspace from a convenience to a necessity, occupying an unprecedented place as a means of disseminating information on the pandemic and its effects on the world, ^1^ but also potentially leaving it as the only remaining vector for maintaining social interactions. ^4^

From a technological point of view, the COVID-19 pandemic has caused massive and immediate changes in the use of digital media. ^5^ For the vast majority, use of DT is healthy when practiced in moderation, but for some individuals it can compromise interactions and habitual social activities and other activities of daily living. ^1^ Use of psychoactive substances and other reinforcing behaviors, such as playing videogames, watching TV series, using social networks (SN), consuming pornography, or surfing the internet are often used to reduce stress and anxiety and/or relieve depressed mood. ^3^ Since sources of recreation are limited to the domestic environment, the internet and television provide fertile ground for individuals to develop compulsive behaviors, because these sources are readily accessible, ^6^ and opportunistic marketing campaigns can encourage customers to spend more time in activities that can cause addiction. ^1^ Furthermore, the constant search for information on COVID-19 itself can become an obsession, a phenomenon called “cyberchondria.” ^7^ It is thus necessary to study use of DT as compensation for the pandemic’s effects, plus the positive and negative behavioral impacts ^2^ and the support systems and digital infrastructure needed for the current and future pandemics. ^8^

This study aims to validate the Behavioral Changes Scale on the Use of Digital Technologies during Social Distancing (BCSDTSD) in the COVID-19 pandemic. The current study focuses on adaptation of daily living to the virtual world, when the population is largely confined to home and with outside circulation severely curtailed. The result is increased hours using technological devices, expansion and diversification of digital activities, adaptation of consumption of products and services via the internet, social and professional relations limited to videoconferencing, and physical and emotional changes associated with this new lifestyle. In this case, no similar preexisting scale was found.

## Method

### Research

Research was conducted searching the PubMed, ISI Web, and SciELO databases, by combining variants of keywords: digital technologies, internet, social distancing, scale, and COVID-19. Several scales were examined to support composition of BCSDTSD classification items and structures, merging different domains of mental health (MH) that may be affected by SD, thus allowing assessment of individual and collective behavior in response to the pandemic. These scales include: the Abusive Use of Technologies Scale (TAUS) ^9^ ; the Cell Phone Dependency Scale (CPDS) ^10^ ; the Facebook Dependence Scale (FDS) ^11^ ; the WhatsApp Dependence Scale (WADS) ^12^ ; the Digital Dependence of Employees Scale (DDES) ^13^ ; the scale to assess Pathological Digital Game Dependence (PDGD) ^14^ ; the Physical Damages related to the Abusive Use of Technology Scale (PDAUTS) ^15^ ; and the Technology Dependent Depression Scale (TDDS). ^16^

### Construction of the BCSDTSD

Validation of the BCSDTSD instrument comprised five phases: 1. construction of a preliminary scale with 10 questions; 2. evaluation of the questions by a panel of experts; 3. administration of the scale to 1,012 volunteers in SD; 4. statistical analysis of the results; and 5. preparation of the final version of the validated scale.

The present study consulted three experts (PhD professors) to validate the BCSDTSD scale, who evaluated the instrument’s adequacy in terms of clarity, alignment with the research objective, and coherence between the questions. The experts approved a final version of the scale for administration comprising the same 10 original questions.

According to Hair, ^17^ a questionnaire should not be administered before the researcher assesses the likely accuracy and consistency of the answers. A valid scale requires that the content is developed fully in accordance with the respective theme and with the study’s objectives, after which it is submitted to a group of faculty and physicians with expertise in the subject (digital dependence [DD] in this case). ^13^ There is no consensus on the number of experts who should participate in scale validation, which is thus left to the researcher’s discretion and accessibility. ^10^

This instrument, consisting of just 10 questions and featuring direct and simplified messages, aims to maximize respondents’ capacity for action and attention. ^4^ Each item is responded on a three-point scale that expresses the degree to which the individual agrees with the statement, ^18^ resulting in a final score for classification with the scale. The 10 questions in the BCSDTSD instrument address mild, moderate, and severe changes in individuals’ lives resulting from the use of DT during SD. The response values were: no (0); yes, a little (1); and yes, very much (2) to verify the intensity of changes in behavior. This division into responses with different intensities was adopted from all the scales examined when constructing the BCSDTSD, although in those scales the classification was divided into four categories. For the BCSDTSD the number of response categories was reduced to three because the questionnaire was restricted to just 10 items, while the other scales contained from 16 to 20 questions.

### Sample

Following validation by the experts, the instrument was ready for administration to its respective target audience, who were recruited randomly through open invitation via Facebook, LinkedIn, and direct mail. The sample comprised 1,012 participants of both sexes and various age groups.

Participants’ anonymity was guaranteed and the study did not involve individual interventions and did not pose any risk to the subjects. The study analyzed the statistical results for the sample of individuals as a whole and not the specific answers of individual participants.

### Ethical standards

All procedures performed in studies involving human participants were in accordance with the ethical standards of the institutional and/or national research committee and with the 1964 Declaration of Helsinki and its later amendments or comparable ethical standards. The study was conducted with integrity and with ethical principles and approved by the research ethics committee at the Instituto de Psiquiatria, Universidade Federal do Rio de Janeiro (CAAE: 29048920.1.0000.5263).

### Data collection

Data were collected from May 6 to June 13, 2020, via the internet, using the Google Forms app, widely adopted in similar studies. The results of data collection were keyed into a database created in Excel from the application used, avoiding possible typographical errors by the researcher. The data then underwent statistical analysis to validate the scale and to characterize the sample profile according to its demographic data.

### Inclusion and exclusion criteria

Inclusion criteria were individuals over 18 years of age who had their daily activities limited by isolation at home during the study period. Individuals whose work activities prevented them from remaining in total SD were excluded.

### Data analysis

The criteria used to validate the scale were: chi-square tests, to identify relations between the questions on the scale and the demographic data of the sample; Bartlett’s test of sphericity, to perform the factor analysis; the Kaiser-Meyer-Olkin (KMO) ^19^ statistic, to verify the adequacy of the factor analysis; the proportion of variance method, to determine the number of relevant factors; and Cronbach’s alpha, to measure the questionnaire’s internal consistency. Data were analyzed using the *dplyr* , ^20^ *psy* , ^21^ and *paran* ^22^ packages and the REdaS software program. ^23^ These were the same criteria used in the scales examined, the only difference being the presence of control groups for comparison to the respective groups evaluated. In the case of BCSDTSD, this was not possible, because the evaluation was unprecedented in history. In this case, there are no control groups for comparison.

## Results

From the initial sample, 975 participants’ data were analyzed and 37 questionnaires that were not fully completed were excluded, since none of the questions were mandatory. Table 1 provides a summary of the sample.

**Table 1:** Sample characteristics

Characteristics	
Gender	
Male	336 (34)
Female	639 (66)
Age brackets (years)	
18-25	50 (5)
26-33	104 (11)
34-41	161 (17)
42-49	175 (18)
50-57	212 (22)
58-65	158 (16)
66-70	115 (12)

Data presented as n (%).

Anshari et al. ^24^ had already concluded that gender is statistically significant in use of smartphones, with women presenting more accentuated use of SN and of sharing text messages. In addition, women are more susceptible to smartphone dependence ^25^ and nomophobia. ^26^ These references may explain the participation of almost twice as many female volunteers (66%) than men (34%) in this research.

The classification of age brackets (AB) began at 18 years of age, as this is officially considered entry into adulthood, due to the insertion of a significant portion of these individuals in the labor market. ^27^ The National Youth Policy (Política Nacional de Juventude [PNJ]) in Brazil classifies the AB from 18 to 24 years old as young people, since a good part of them reconcile work with studies, and people aged 25 to 29 years as young adults, ^28^ since the majority of the members of this AB are considered adult and independent. ^27^ These demographic criteria are important premises for characterization of the young population, but insufficient for investigations of segments over 25 years old. ^27^ Thus, the first AB of the study was delimited as 18-25, serving as a model for all subsequent ABs, at 7 years each, with the exception of the last AB. Despite the small research bias involved, the last group is restricted to 70 years of age, because that is when the proportion of aging considered pathological increases, i.e., aging associated with diseases and disabilities. ^29^

Kachar ^30^ points out that ABs over 45 years old show a higher propensity to search for information related to health, goods, and services, which would explain the greater participation of people between 42 and 57 years old in a study related to DT and the COVID-19 pandemic. His hypothesis for the drop in the percentage of participation of those over 60 years old has to do with the lack of interest and/or knowledge about all the possibilities that can be accessed with technological resources. ^30^

The chi-square test performed to verify whether the questionnaire responses are related to gender indicated that only questions 1 (p-value = 0.160), 6 (p-value = 0.090), and 10 (p-value = 0.446 ) are not related, i.e., there were no behavioral differences between men and women regarding the increase in connected hours (Q1), videoconference with family and friends (Q6), or control of routine well-being (Q10). Application of the same test to ABs showed that only questions 1 (p-value = 0.400) and 3 (p-value = 0.101) were not related to age. The frequency of usage at least once a week is similar among all ABs ^30^ and so is use of smartphones for conversation, ^30^ explaining the almost unanimous increase in hours of use due to distancing (Q1). The same occurs with adaptation of consumption habits (Q3). Thus, we can say that the different genders and ABs do not have the same behavior in most questions on the scale.

In order to perform the factor analysis, Bartlett’s test of sphericity was performed to verify whether the variables were correlated with each other. The null hypothesis in this test is that the correlation matrix is equal to the identity matrix. The data set showed a test statistic of 1632.81 and a p-value less than 2.22e-16, implying that the covariance matrix was not equal to identity.

The next criterion used to verify adequacy for factor analysis was the KMO ^19^ statistic. The resulting value was 0.795, very close to 0.8, considered good, ^19^ and the Measure of Sampling Adequacy (MSA) ^19^ for each of the variables was greater than 0.6.

The results of both Bartlett’s test and the KMO support conducting factor analysis on the questionnaire. The low p-value in Bartlett’s test of sphericity indicates correlation between the variables, and the KMO statistic confirms the adequacy for factor analysis, with nine of the 10 items on the scale yielding values greater than 0.7, considered satisfactory. ^19^

The next step was to check the factor loads to determine the number of relevant factors. The method employed was proportion of variance, that is, factors were only maintained when their combined variance accounted for more than 90% of the total variance. To meet this criterion, eight factors must be used, and, in this case, the commonality was greater than 74% for each variable. The commonalities showed that no questions had to be eliminated from the questionnaire, since all values were greater than 0.5.

The last step in the study was to calculate Cronbach’s alpha using the *psy* package, in order to measure the questionnaire’s internal consistency. The resulting value was 0.725, considered satisfactory for validation of a new scale. ^31^

The dataset described here was satisfactory, considering the number of items on the initial scale (10). The assessment of changes in digital behavior was necessarily based on participants’ own perception, due to the lack of validated measures for comparison and the imposition of unprecedented SD in the digital age. ^5^ Although measures based on participants’ perceptions have been used previously and given the relevance of perceived SD for health and the adaptation of basic daily needs, we do not know how these perceptions align with objective digital behavior. ^32^

The valid questionnaires employed in the analyses were quantitatively consistent and the demographic data did not seem to affect the response pattern of the BCSDTSD.

## Discussion

Although SD is a traditional public health measure, this is the first time that it has been imposed on such a large proportion of the world population ^33^ and on healthy individuals, since previously only the sick and/or those exposed to contagion were isolated. ^2^ One major difference in this pandemic is precisely the presence of the internet, which is why Yan ^2^ suggests studies of the types of people who started using emerging technologies, which applications and DT were used, the new human-technology interactions, and what behavioral impacts resulted, proposing that this is how to understand how humans behave with DT, both in extreme and in common events. Silva et al. ^34^ considers that relevant issues are sometimes omitted within the limitations of statistics and research done on the internet, such as the time spent on each online practice, hours of use of DT, type of content accessed/shared, which services platforms and applications, and also who the disconnected people are. These observations and lines of study are consistent with the BCSDTSD, paying attention to ordinary individuals subjected to quarantine and their respective behavioral changes in technology use, whereas most scales related to MH created during the pandemic address issues of anxiety and fear of COVID-19 and the effects on infected people and on the health professionals working to combat the pandemic.

The prolonged duration of quarantine favors boredom, frustration, and concern about lack of basic supplies and about financial loss. ^35^ The long-term impact is considerable and wide-ranging, including anxiety, depression, anger, post-traumatic stress symptoms, ^36^ abuse of alcohol, tobacco, and other drugs, changes in sleep and/or eating patterns, difficulty concentrating, ^37^ mental confusion, ^35^ and behavioral changes, such as avoiding crowded places and washing hands excessively. ^36^ These psychological symptoms can last up to three years after the quarantine period. ^38^ It is thus predicted that there will be increases in mental disorders (MD) and psychiatric illnesses after the pandemic and that the impact on MH may even be lasting. ^36^ A deterioration in the MH of the Hong Kong population has been documented, with increases in the prevalence rate of suspected depression to 11.2% and of post-traumatic stress disorder to 12.8%. ^39^ Studies with different populations have shown high levels of psychological distress in Spain (72%) and high prevalence of depression (24.7%) and anxiety (23.2%) in Italy and North America (44.1 and 47.2%, respectively). ^33^ In the United Kingdom, mental distress increased from 18.9 to 27.3% in just one month of lockdown. ^40^ Passos et al. ^33^ conducted a cross-sectional study in on the impacts on the MH of adults in Brazil and Portugal during the pandemic and the results corroborate those of previous studies: the prevalence of anxiety in the sample was 71.3%, 24.7% of the sample had depression, and 23.8% had both depression and anxiety. The observed frequencies were considerably higher than pre-COVID-19 levels. Even before the pandemic, Brazil already had the highest prevalence of anxiety of any country in the world, with 9.3% of the population showing some type of anxiety disorder. ^33^ Campos et al. ^40^ conducted a study of the psychological impact of SD due to the COVID-19 pandemic on 13,584 volunteers from all regions of Brazil. The results also prove the high prevalence rates of depression (61.3%), anxiety (44.2%), stress (50.8%), avoidance (59.2%), intrusion (46.8%), hyperexcitation (50,1%), and psychological impact (54.9%). ^40^ Approximately 88.8% of the sample presented some new symptom after the start of the pandemic, with rates of 85.5% among individuals who had no previous diagnosis and 96.2% among those with prior diagnoses. ^40^ Those most susceptible to developing psychological symptoms are young people, women, people with lower economic status, those excessively exposed to the news, those who felt insecure, and those with a previous diagnosis of MH and/or who had general health problems before the pandemic. ^40^ It can therefore be suggested that the COVID-19 pandemic significantly affected the MH of the adult population in Brazil.

Technological devices complement daily activities and offer several opportunities for expanding new horizons of information, professional alternatives, leisure-time activities, personal contacts, and numerous ways to alleviate loneliness and boredom. ^41^ A study by Wiederhold ^42^ on the use of SN during SD reports a considerable increase in use of television, with viewing time typically skyrocketing during major disasters and a sharp 20% increase in web traffic between 8 and 15 March, 2020, a period in which the World Health Organization (WHO) established SD as a preventive measure worldwide. The Facebook and Twitter platforms quickly witnessed a major increase in user traffic, ^42^ the volume of downloads and use of online game data reached record highs, ^43^ there was a global increase of 11.6% in use of virtual pornography, ^44^ and 30.8% of the population reported that they watched videos on television or online to keep up-to-date. ^6^ In 2016, the Brazilian Institute of Geography and Statistics (Instituto Brasileiro de Geografia e Estatística [IBGE]) reported that 64.7% of the Brazilian population was connected to the internet. The 2019 data show a considerable increase, at 71%, and with 64% of this group using some SN. ^45^ It is assumed that these numbers will reach even higher levels after the start of SD. Abusive use of digital spaces can lead to potentially compulsive exposures, ^4^ such as digital gaming (DG), compulsive shopping, overuse of SN, and pornography, reinforcing digital vicious cycles. ^1^

Disordered use of the internet generates marked distress and/or significant damage in the personal, social, educational, and occupational areas. ^1^ A good example of a lack of control in activities associated with technologies is DG, which, although it may be an adaptive strategy to deal with the pandemic, can evolve into long-term DD. ^43^ In general, digital addicts do not see themselves as such, especially because they deal with materials and activities that are widely used in modern times. ^13^ Mescollotto et al. ^46^ assessed 130 young Brazilians for smartphone dependence and the results showed that 33.1% were addicted, with an average use of 5 hours/day, and also showed high rates of wrist pain (31.4%) and cervical pain (44.6%) among volunteers. These musculoskeletal changes demonstrate that a lack of guidance on correct use of DT can have serious physical consequences, ^15^ in addition to the emotional damage already reported. This is one of the reasons for the concern with constructing the BCSDTSD for assessment of these physical and emotional perceptions and, mainly, measuring the possibility of DD among the respondents. After all, the data from the study by Mescollotto et al. ^46^ are alarming, although they are minimized by the subjectivity of the participants, since the average time recorded represents almost 21% of the day using a device, even among those who were not dependent. Another key issue is the expected increase in new technology-dependent habits during SD that may persist after the pandemic, such as online shopping, food delivery, social interaction exclusively by DT, courses, and videoconference consultations. ^47^ It is with regard to these issues that the BCSDTSD scale is particularly important, since its questions 2 to 7 deal with these variations in technological activities.

Although SD is not necessarily synonymous with loneliness, early indications in the context of COVID-19 indicate that almost half of young people between 18 and 24 years of age are lonely during confinement. ^35^ Loneliness is also related to Hikikomori syndrome (HS), because, although it is not one of its mandatory diagnostic criteria, it is a very pronounced characteristic with continuous social withdrawal ^47^ and also a fundamental basis for the construction of an evaluation scale. ^48^ HS is currently seen as a socio-cultural phenomenon of MH, rather than a typical mental illness, characterized by prolonged and severe social withdrawal for a period of at least 6 months. ^49^ Prevalence of illness estimates in Asian community populations range from 0.87 to 2.3%. ^47^ In Brazil, prevalence is still unknown due to the lack of empirical research on the phenomenon, although there are already three reports in Brazilian patients. ^47^

Tateno et al. ^50^ demonstrated the strong relationship between HS and DD when verifying that individuals with high risk scores on the Hikikomori Questionnaire (HQ-25) spent more time using the internet and had higher scores on the Internet Addiction Test (IAT) and on the Smartphone Addiction Scale - Short Version (SAS-SV). He further noted that as social media apps are becoming more popular, users are more connected to the internet and the time spent with people in the real world continues to decrease. ^50^ Aspiring to social death and avoiding physical death is a central feature of people with HS, so many of them will continue to passively observe the world through online DG and SN, as long as their parents ensure that the basic needs of their lives are met. ^51^

Economic, social, or political crises can cause even previously healthy people to enter a state of Hikikomori with psychiatric conditions in the post-pandemic world. ^52^ Kato et al. ^52^ states that individuals who experience SD related to the pandemic can be measured using the same scale as individuals with Hikikomori, despite the isolation of the first group being imposed by government restrictions and/or due to fear of infection by the coronavirus. Transnational studies of HS show that, without intervention, the period of social abstinence can last for years and, in some cases, the entire adult life.

Thus, COVID-19 also makes it more likely that serious damage to the MH of individuals will occur, which may last way beyond resolution of the pandemic. ^53^

One limitation of this study was the absence of other specific validated instruments for investigating behavioral changes in the use of DT during SD, which could have been used in constructing this scale. However, the methodological rigor dedicated to construction of the scale and the confirmation of the 10 questions proposed initially ratify the instrument’s quality.

We recommend further studies on digital behavior and its consequences during quarantine periods, since DT are essential for dealing with crises like the COVID-19 pandemic. The internet can enable maintenance of the necessary social boundaries for people to remain “alone together” during the pandemic, as well as to compensate for the closing of schools and universities, thereby reducing some of the impacts of the economic crisis through remote work (home office), ^5^ disseminating reliable information on disease prevention and containment in real time, ^53^ and even facilitating medical, psychological, and psychotherapeutic consultations. Future studies should also address the influence of DT on children ^15^ and inclusion of individuals and groups currently excluded from digital technologies, whether due to digital illiteracy, lack of connectivity in remote locations, or the impossibility of accessing technological devices. ^4^ It is essential to find creative ways to extend the reach of information technology to all aspects of society, including work and personal life. ^8^ It is thus necessary to unveil this comprehensive universe in order to plan preventative measures, treat disorders that have already developed, reduce future harm, and identify and propose solutions to the problems raised by this health crisis in our modern world.

## Conclusion

The study achieved its objective of presenting a validated final version of the BCSDTSD. The statistical results showed that the 10 questions in the initial version were aligned with each other and could be maintained as valid and relevant for assessing individuals’ perception of behavioral changes related to interaction with technological devices during the SD with COVID-19 pandemic. Development of the BCSDTSD instrument is thus justified for observing changes and impacts in individuals’ routines under conditions of isolation.
